# Rapid First-Year Proximal Femoral Bone Loss After Traumatic Transfemoral Amputation and Its Partial Reversal With Sequential Romosozumab and Antiresorptive Therapy: A Case Report

**DOI:** 10.7759/cureus.116980

**Published:** 2026-09-26

**Authors:** Mitsunori Toda, Takashi Oshima

**Affiliations:** 1 Orthopaedic Surgery, Rehabilitation Medicine, Hyogo Prefectural Rehabilitation Center Central Hospital, Kobe, JPN; 2 Orthopaedic Surgery, Hyogo Prefectural Rehabilitation Center Central Hospital, Kobe, JPN

**Keywords:** amputee, bone mineral density, disuse osteoporosis, dual-energy x-ray absorptiometry, prosthetic rehabilitation, romosozumab, sclerostin, secondary osteoporosis, sequential therapy, transfemoral amputation

## Abstract

Lower limb amputation causes early loss of bone mineral density (BMD) in the residual limb, and the loss is greater after transfemoral than after transtibial amputation. Densitometry is nevertheless rarely performed after amputation, and no report has described pharmacological treatment of this bone loss. A 37-year-old man underwent right transfemoral amputation after a road traffic injury; a concomitant left femoral shaft fracture was treated with an intramedullary nail. He was admitted to our hospital five months after the injury for prosthetic fitting and gait training, and dual-energy X-ray absorptiometry (DXA) was performed for the first time at that point. Three months later, during intensive inpatient gait training with progressive weight bearing, BMD had decreased at every site, and the magnitude of the decrease was graded according to loading condition: 1.9% at the lumbar spine, 8.7% at the non-amputated femoral neck, 15.6% at the amputated femoral neck, and 20.1% at the amputated total hip. Romosozumab 210 mg monthly was administered for 12 months, from 9 to 20 months after amputation, followed by denosumab and then zoledronic acid. Over 33 months of sequential treatment, BMD increased at every site relative to the value at treatment initiation, by 32.6% at the lumbar spine and 33.2% at the amputated femoral neck, but recovery was incomplete: BMD at the amputated total hip remained 2.5% below the value recorded five months after amputation. No fracture occurred, and the patient remains in employment. Rapid proximal femoral bone loss can therefore progress after transfemoral amputation despite prosthetic gait training and daily prosthesis use. This is an uncontrolled observation in a single patient: fracture healing, continuing rehabilitation, and progressively increasing physical activity coincided with pharmacotherapy, and the increase in BMD cannot be attributed to the medication. Whether early and repeated densitometry after lower limb amputation identifies this decline while it remains treatable and whether treatment reduces fractures require prospective evaluation.

## Introduction

Reduced bone mineral density (BMD) is a well-recognized consequence of major lower limb amputation, and the deficit is greater after transfemoral than after transtibial amputation [1]. In a prospective study of eight traumatic amputees enrolled before surgery, areal BMD at the hip of the amputated limb decreased by 11-15% and had not recovered after 12 months of prosthetic ambulation; volumetric BMD and estimated bone strength in the residual bone decreased further still, and the authors identified the early post-amputation period as the most critical window for preventing bone loss [2]. Cross-sectional data indicate that the deficit is regional rather than systemic. In the Armed Services Trauma Rehabilitation Outcome (ADVANCE) study, which compared 575 men with combat-related traumatic injury with 562 matched uninjured men, the reduction was confined to the femoral neck of the amputated limb, was greater with more proximal amputation, and was not accompanied by any difference in spine BMD or activity level [3]. After transfemoral amputation, an ischial containment socket transfers body weight to the ischium and pubic ramus rather than through the femoral shaft, so that the residual femur receives the bending and torsional loads generated by the hip musculature but little axial load.

These changes have clinical consequences. People with lower limb amputation fall more frequently than able-bodied individuals at every stage of recovery, and the risk is highest during the first four years after amputation [4]. Femoral fracture in this population is most often sustained on the amputated side, and hip fracture in an amputee carries higher mortality than hip fracture in a comparable non-amputee cohort [5]. Although the published series consist predominantly of elderly patients, with a mean age of 78 years in one retrospective series [5], proximal femoral fracture after a minor indoor fall also occurs in working-age transfemoral amputees who walk independently with a prosthesis and are in employment [6]. For such a patient, the fracture threatens not only walking but also employment and community participation, which are the ultimate goals of prosthetic rehabilitation.

Interventions proposed to counter this bone loss have so far relied on mechanical loading. An international Delphi panel has published consensus recommendations on exercise prescription to minimize hip BMD loss after traumatic lower limb amputation, while noting that these recommendations have not been tested in clinical practice [7]. We have previously reported a young man who, after transtibial amputation, undertook a rehabilitation program incorporating high-impact and resistance training and showed a 17.4%-24.3% increase in BMD of the amputated-side hip over three years [8]. Whether a comparable response can be obtained after transfemoral amputation, in which an ischial containment socket transfers load to the pelvis and largely bypasses the residual femur, is unknown; reduced BMD at the impaired hip has been reported even in amputee athletes, indicating that a high level of physical activity does not by itself prevent site-specific bone loss [9]. Pharmacological intervention for unloading-related bone loss has been investigated principally in spinal cord injury: in women with chronic spinal cord injury, 12 months of romosozumab increased areal BMD at the lumbar spine and total hip [10]. To our knowledge, no published report has addressed pharmacological treatment of the bone loss that follows lower limb amputation.

This report describes a 37-year-old man with traumatic transfemoral amputation in whom serial dual-energy X-ray absorptiometry (DXA) documented a 20.1% decrease in BMD at the total hip of the amputated side over three months within the first year after amputation, during intensive prosthetic gait training with progressive weight bearing, and in whom sequential treatment with romosozumab, denosumab, and zoledronic acid was followed by substantial but incomplete recovery over the subsequent 33 months.

## Case presentation

This study was approved by the Institutional Review Board of Hyogo Prefectural Rehabilitation Center Central Hospital (approval number 1906), and the patient gave written informed consent to participate and to the use and publication of the anonymized clinical details, laboratory data, and radiographic images contained in this report.

Initial injury and acute management

A 37-year-old man, employed as a security guard, was struck from behind by a passenger car while working on a road median strip. At a tertiary emergency center, he was diagnosed with an open fracture of the right knee joint, an open fracture of the left femoral shaft, a fracture of the left fibula, and bilateral pubic rami fractures. The soft tissues around the right knee were severely crushed, and the limb was not salvageable; right transfemoral amputation with stump plasty was performed on the day of injury. Four days later, the left femoral shaft fracture was treated by open reduction and internal fixation with an antegrade intramedullary nail. The remaining fractures were managed conservatively. The injury was sustained at work and was covered by the national workers’ compensation scheme. The clinical course from injury to the final assessment is summarized in Figure 1.

**Figure 1:**
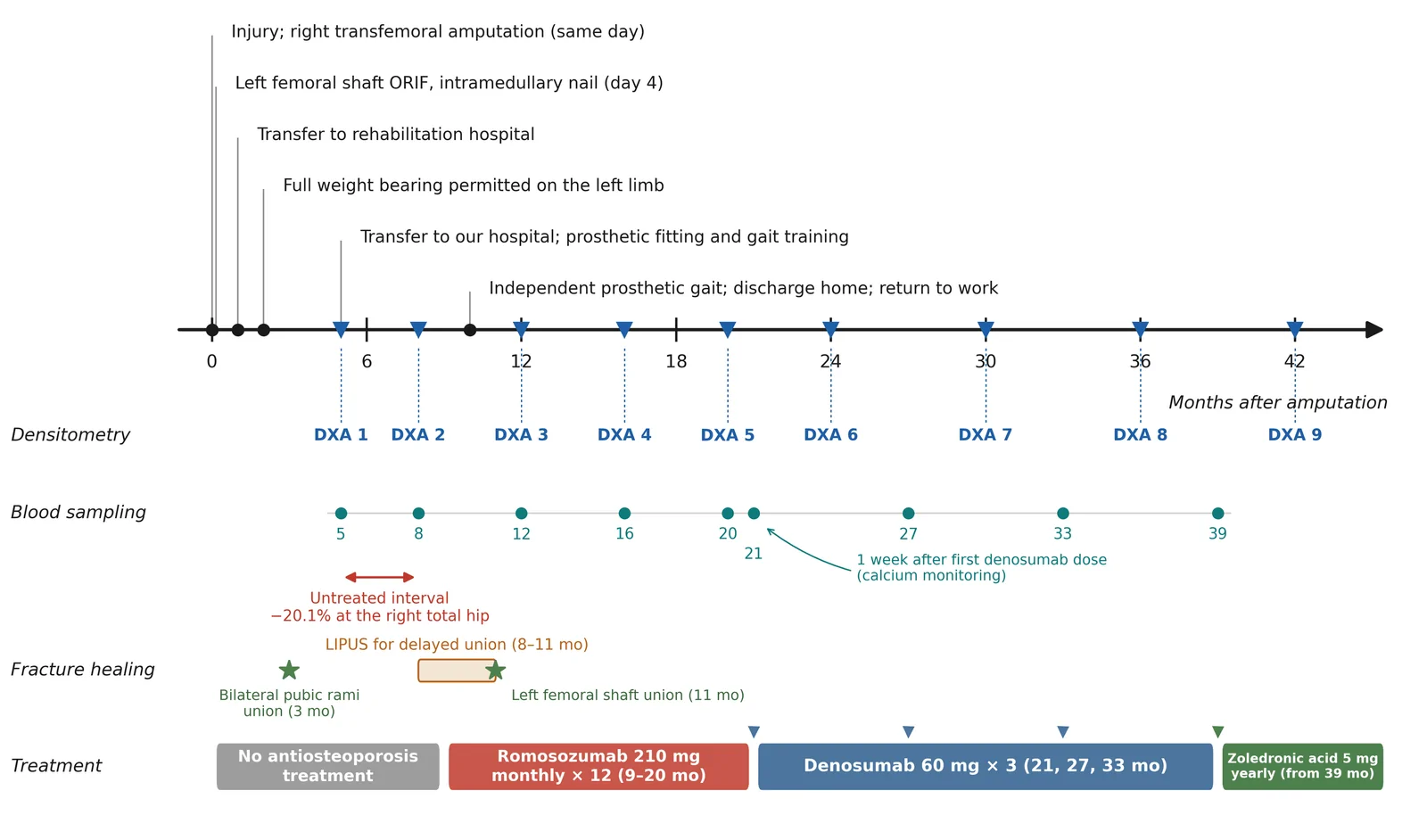
Clinical course from injury to the final assessment. The upper rows show the clinical events. The main axis gives the time in months after amputation; the amputation was performed on the day of injury. Blue inverted triangles mark the nine DXA examinations at 5, 8, 12, 16, 20, 24, 30, 36, and 42 months, and the row below shows the nine occasions on which blood was sampled at 5, 8, 12, 16, 20, 21, 27, 33, and 39 months. The two sets of visits do not coincide because blood was drawn on the days of drug administration, whereas densitometry was performed at the intervals at which it can be claimed under national health insurance. The sample at 21 months was obtained one week after the first denosumab dose as part of routine monitoring for hypocalcemia after the initial administration. The red arrow marks the interval between the first two examinations, during which no treatment for bone loss was given and BMD at the right total hip decreased by 20.1%; this interval coincided with intensive inpatient prosthetic gait training. The fracture-healing row shows union of the bilateral pubic rami at 3 months, the period during which low-intensity pulsed ultrasound (LIPUS) was applied to the left femoral shaft for delayed union, from 8 to 11 months, and union of that fracture at 11 months. The treatment row shows the period before antiosteoporosis treatment, romosozumab 210 mg monthly from 9 to 20 months, denosumab 60 mg at 21, 27, and 33 months, and zoledronic acid 5 mg from 39 months; the small inverted triangles above the bars mark the individual antiresorptive administrations. The LIPUS shown in the fracture-healing row was directed at the left femoral shaft and overlapped the first two months of romosozumab treatment. BMD, bone mineral density; DXA, dual-energy X-ray absorptiometry; LIPUS, low-intensity pulsed ultrasound; ORIF, open reduction and internal fixation.

Height, measured at admission to our hospital without the prosthesis in single-limb stance with handrail support, was 163 cm, and body weight, also measured without the prosthesis, was 54 kg, giving an uncorrected body mass index of 20.3 kg/m^2^. Because measured body weight underestimates adiposity after lower limb amputation, a corrected value was calculated as measured weight divided by (1 − the proportion of body mass of the absent segment), using published segmental proportions (below-knee segment with foot, 5.9% of body mass; thigh, 10.1%) and estimating the absent proportion of the thigh from the ratio of residual to contralateral femoral length (35 cm and 42 cm, respectively) [11]; the corrected body mass index was 22.0 kg/m^2^. No notable premorbid medical history was documented, and he had not been taking any medication known to affect bone metabolism before the injury. He had no diabetes mellitus (glycated hemoglobin 5.6%), and renal function was normal (estimated glomerular filtration rate 104 mL/min/1.73 m^2^).

Rehabilitation and prosthetic fitting

One month after the injury, he was transferred to a regional rehabilitation hospital. Full weight bearing on the left lower limb was permitted eight weeks after internal fixation. He became independent in basic activities of daily living at wheelchair level, and standing and weight-bearing training of the left lower limb was performed within parallel bars with upper-limb support. Disuse of the left lower limb was nevertheless marked, and recovery was slow.

Five months after the injury, he was transferred to our hospital for prosthetic fabrication and gait training. On admission, he mobilized in a standard wheelchair. The residual limb was 35 cm long (83% of the contralateral limb length of 42 cm) and cylindrical; the surgical wound had healed; and there was no joint contracture. Manual muscle testing of the residual limb was grade 4 for hip flexion, extension, abduction, and adduction. Single-limb stance on the left was possible with one-hand support and could be maintained for approximately 10 seconds without support. Phantom limb sensation and phantom limb pain were both present. Pregabalin was titrated gradually to 150 mg daily; further escalation was limited by somnolence and by concern about the associated risk of falling, and the patient elected to continue at this dose. He lived alone on the second floor of an apartment building without an elevator and had no family support available; his workplace supervisor acted as his key contact person. The bilateral pubic rami fractures had united three months after injury. The left femoral shaft fracture was not yet united: callus was visible radiographically and weight bearing was possible, but cortical continuity remained incomplete in part of the fracture.

A transfemoral prosthesis was prescribed, comprising an ischial ramal containment suction socket, a Hybrid Knee prosthetic knee (Nabtesco, Kobe, Japan), and an FS3000 prosthetic foot (Nabtesco). After five months of intensive prosthetic rehabilitation, he achieved independent prosthetic gait indoors and outdoors without walking aids. He was discharged home 10 months after the injury and returned to work in a clerical position. His functional level thereafter corresponded to Medicare Functional Classification Level K3 [12]; this was a clinical estimate and was not determined with a quantitative instrument such as the Amputee Mobility Predictor. He wore the prosthesis every day from 07:00 to 20:00, approximately 13 hours daily.

The left femoral shaft fracture continued to heal slowly during prosthetic rehabilitation. Radiographic follow-up at eight months after injury showed union to remain inadequate; delayed union was diagnosed, and low-intensity pulsed ultrasound (LIPUS) was applied to the fracture site until complete radiographic union was confirmed at 11 months. The intramedullary nail was not removed. At no point was prosthetic gait training restricted on account of the fracture.

Bone mineral density at the first assessment

All nine DXA examinations were performed on the same densitometer (Discovery; Hologic, Marlborough, Massachusetts) in the same department, using the routine hip acquisition and analysis procedure of our institution. No protocol specific to the amputated limb had been established. The patient was scanned supine without the prosthesis, and the residual limb was not stabilized with a dedicated positioning device; because internal rotation cannot be set through the foot after transfemoral amputation, the position of the residual limb was judged visually by the radiographer at each examination and was not standardized between examinations. The manufacturer's default region of interest was applied and was not manually resized. All scans were acquired by the same group of radiographers and were analyzed by the same operator. The first examination was carried out at the time of transfer to our hospital, five months after the amputation (Table 1).

**Table 1:** Serial dual-energy X-ray absorptiometry measurements. BMD is expressed in g/cm^2^. The right side is the amputated side. BMD of the left total hip and left trochanter could not be measured because the intramedullary nail used for fixation of the left femoral shaft fracture was included in the region of interest; only the left femoral neck was assessable. All scans were performed on the same densitometer. The percentage of the young adult mean (YAM) was provided by the densitometer only for the lumbar spine and femoral neck and is therefore not tabulated; the YAM values cited in the text refer to the right femoral neck. BMD, bone mineral density; YAM, young adult mean; ROMO, romosozumab 210 mg subcutaneously every month from 9 to 20 months after amputation; DMAB, denosumab 60 mg subcutaneously at 21, 27, and 33 months; ZOL, zoledronic acid 5 mg intravenously from 39 months. The examinations listed here are densitometry visits; blood was sampled at different visits (Table 2).

Parameter	Category	Months after amputation								
		5	8	12	16	20	24	30	36	42
Months relative to romosozumab		−4	−1	+3	+7	+11	+15	+21	+27	+33
Treatment		None	None	ROMO	ROMO	ROMO	DMAB	DMAB	DMAB	ZOL
Lumbar spine (L2–L4)	BMD	0.810	0.795	0.882	0.957	0.976	1.028	1.046	1.066	1.054
	T-score	−2.0	−2.1	−1.4	−0.8	−0.6	−0.2	0.0	0.2	0.1
	Z-score	−1.5	−1.6	−1.0	−0.4	−0.3	0.2	0.2	0.4	0.2
Left femoral neck (non-amputated)	BMD	0.647	0.591	0.641	0.657	0.682	0.712	0.706	0.704	0.733
	T-score	−1.7	−2.1	−1.7	−1.6	−1.4	−1.2	−1.2	−1.2	−1.0
	Z-score	−1.4	−1.9	−1.5	−1.3	−1.1	−0.8	−0.9	−0.9	−0.6
Right femoral neck (amputated)	BMD	0.564	0.476	0.523	0.540	0.554	0.581	0.618	0.613	0.634
	T-score	−2.4	−3.0	−2.7	−2.5	−2.4	−2.2	−1.9	−2.0	−1.8
	Z-score	−2.1	−2.8	−2.4	−2.3	−2.1	−1.9	−1.6	−1.6	−1.5
Right trochanter (amputated)	BMD	0.508	0.434	0.459	0.476	0.491	0.521	0.542	0.545	0.546
	T-score	−2.1	−2.7	−2.5	−2.4	−2.3	−2.0	−1.9	−1.8	−1.8
	Z-score	−1.9	−2.5	−2.3	−2.2	−2.1	−1.8	−1.6	−1.6	−1.6
Right total hip (amputated)	BMD	0.670	0.535	0.544	0.556	0.586	0.593	0.648	0.636	0.653
	T-score	−2.2	−3.2	−3.1	−3.0	−2.8	−2.7	−2.3	−2.4	−2.3
	Z-score	−2.0	−3.0	−2.9	−2.8	−2.6	−2.5	−2.1	−2.2	−2.0

BMD was 0.810 g/cm^2^ at the lumbar spine (T-score −2.0, Z-score −1.5), 0.647 g/cm^2^ at the left (non-amputated) femoral neck (T-score −1.7, Z-score −1.4), and 0.564 g/cm^2^ at the right (amputated) femoral neck (T-score −2.4, Z-score −2.1). BMD at the right trochanter and right total hip was 0.508 and 0.670 g/cm^2^, respectively. On the left side, only the femoral neck could be assessed, because the intramedullary nail lay within the regions of interest for the trochanter and total hip.

Bone formation markers were elevated: total N-terminal propeptide of type I procollagen (P1NP) was 167 µg/L (reference range 18.1-74.1) and total alkaline phosphatase (ALP) was 341 IU/L (reference range 38-113). Tartrate-resistant acid phosphatase 5b (TRACP-5b) was 446 mU/dL (reference range 170-590). Serum calcium and inorganic phosphate were within their reference ranges (Table 2). Investigation before treatment comprised a complete blood count, total protein, albumin, AST, ALT, blood urea nitrogen, creatinine, sodium, potassium, chloride, calcium, inorganic phosphate, TRACP-5b, and total P1NP. Serum 25-hydroxyvitamin D, intact parathyroid hormone, testosterone, LH and FSH, thyroid function, serum protein electrophoresis, urinary calcium, and coeliac serology were not measured. There was no history of hypogonadism or delayed puberty, no glucocorticoid or anticonvulsant use, no malabsorptive symptoms, and no family history of fragility fracture; the patient neither smoked nor drank alcohol. The evaluation for secondary causes was therefore incomplete before the start of potent osteoporosis therapy, which limits interpretation of both the baseline values and the subsequent response. These measurements cannot be added retrospectively: no serum was stored, and obtaining new samples would require additional venepuncture outside the scope of the approved protocol.　

**Table 2:** Serial laboratory findings. Values in bold lie outside the reference range. Blood was drawn on the day of each scheduled drug administration. With one exception the sample was obtained immediately before the injection or infusion, so those values are trough values at the end of a dosing interval; the sample at 21 months was obtained one week after the first denosumab dose, as part of routine monitoring for hypocalcaemia. Densitometry was performed at separate visits (Table 1), determined by the interval at which it can be claimed under national health insurance, so the time points of the two tables do not coincide. Total alkaline phosphatase was measured with the same assay method throughout the observation period and was not measured at the 8-, 12-, and 16-month time points. Oral calcium and vitamin D supplements were administered throughout the treatment period. Serum 25-hydroxyvitamin D and intact parathyroid hormone were not measured. P1NP, N-terminal propeptide of type I procollagen; TRACP-5b, tartrate-resistant acid phosphatase 5b; ALP, alkaline phosphatase; eGFR, estimated glomerular filtration rate; ROMO, romosozumab; DMAB, denosumab; ZOL, zoledronic acid.

Parameters	Months after amputation									
	5	8	12	16	20	21	27	33	39	Reference range
Months relative to romosozumab	−4	−1	+3	+7	+11	+12	+18	+24	+30	
Treatment	None	None	ROMO	ROMO	ROMO	DMAB	DMAB	DMAB	ZOL	
Total P1NP (µg/L)	167	142	103	70.8	60.4	12.3	12.4	17.4	8.2	18.1–74.1
TRACP-5b (mU/dL)	446	446	405	296	266	77	81	194	89	170–590
Total ALP (IU/L)	341	—	—	—	246	179	51	54	66	38–113
Calcium (mg/dL)	9.2	9.2	9.3	9.1	9.5	9.8	10.0	9.5	9.5	8.5–10.5
Inorganic phosphate (mg/dL)	4.3	4.3	3.9	3.3	3.6	3.0	3.6	3.0	2.9	2.5–4.7
Creatinine (mg/dL)	0.68	0.73	0.78	0.80	0.81	0.78	0.80	0.85	0.81	0.6–1.1
eGFR (mL/min/1.73 m^2^)	104	96	90	87	85	89	86	80	85	—

In Japan, primary osteoporosis is diagnosed when areal BMD is 70% or less of the young adult mean (YAM) in the absence of fragility fracture [13]. Although the bone loss in this patient was unloading-related rather than primary, this threshold was adopted as a practical reference for treatment decisions, because no diagnostic criterion specific to amputation-related bone loss has been established. BMD at the right femoral neck had already decreased to 70% or less of the YAM, and pharmacological therapy was therefore proposed; the patient declined treatment at that time.

Rapid decline and initiation of pharmacological treatment

A second DXA examination was performed three months later, eight months after the amputation, while the patient was undergoing intensive inpatient prosthetic gait training with progressive weight bearing. BMD had decreased at every site, and the magnitude of the decrease differed markedly between sites: −1.9% at the lumbar spine, −8.7% at the left femoral neck, −15.6% at the right femoral neck, −14.6% at the right trochanter, and −20.1% at the right total hip (Table 3, Figure 2).

**Table 3:** Percentage change in bone mineral density. Values are percentage changes in bone mineral density. No antiosteoporosis treatment was given between 5 and 8 months after amputation; treatment was started at 9 months, one month after the second examination. Despite 33 months of sequential treatment, bone mineral density of the right total hip did not exceed the value recorded at the first measurement.

Site	5 → 8 months (untreated interval)	8 → 42 months (treated interval)	5 → 42 months (overall)
Lumbar spine (L2–L4)	−1.9	+32.6	+30.1
Left femoral neck (non-amputated)	−8.7	+24.0	+13.3
Right femoral neck (amputated)	−15.6	+33.2	+12.4
Right trochanter (amputated)	−14.6	+25.8	+7.5
Right total hip (amputated)	−20.1	+22.1	−2.5

**Figure 2:**
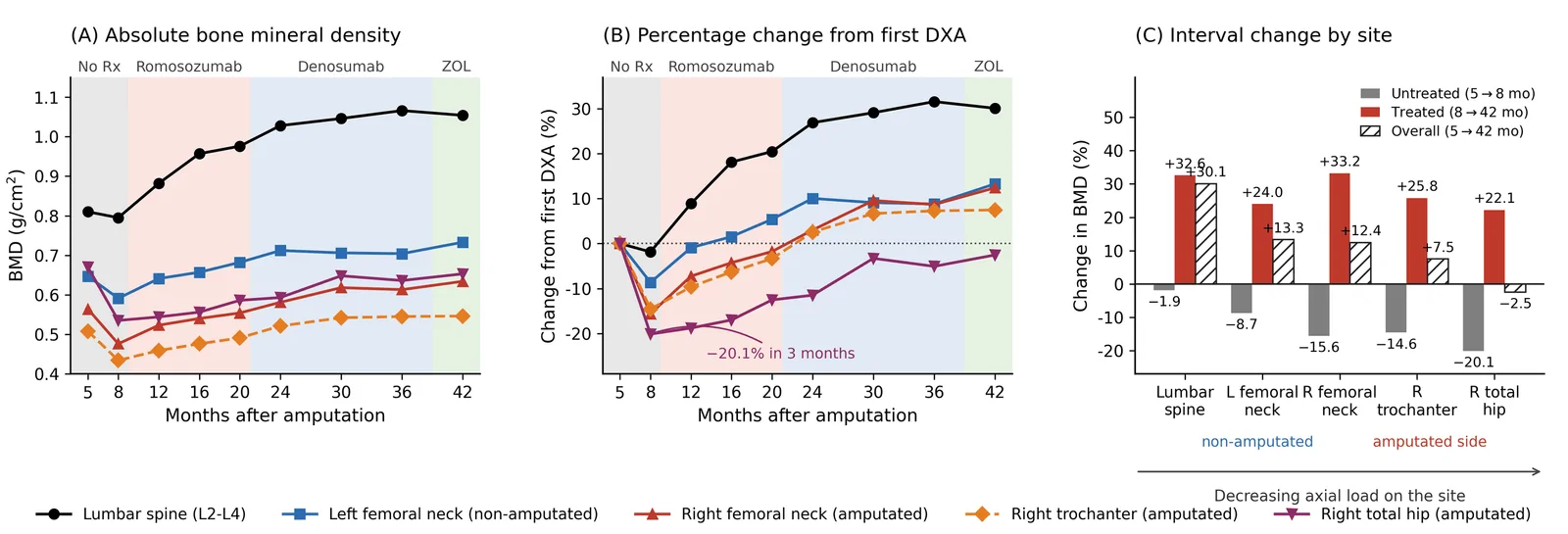
Serial bone mineral density at five measurement sites. (A) Absolute BMD. (B) Percentage change from the first DXA examination. (C) Percentage change in BMD during the untreated interval, from 5 to 8 months after amputation, and during the treated interval, from 8 to 42 months, by site, ordered from the least to the most unloaded site. Shaded bands in (A) and (B) indicate the treatment phases: no treatment until 9 months, romosozumab from 9 to 21 months, denosumab from 21 to 39 months, and zoledronic acid from 39 months. Because densitometry and drug administration were not carried out at the same visits, the boundaries of the shaded bands fall between measurement points. BMD decreased at every site during the untreated interval, with the magnitude of loss graded by loading condition, and increased at every site during sequential treatment. BMD at the amputated total hip did not return to the value recorded at the first examination. BMD, bone mineral density; DXA, dual-energy X-ray absorptiometry; ZOL, zoledronic acid.

The Z-score of the right total hip decreased from −2.0 to −3.0 (Table 1), and BMD of the right femoral neck decreased to 55% of the YAM.

Plain radiographs of the amputated side were obtained at the time of each of these two DXA examinations, both before any pharmacological treatment (Figure 3).

**Figure 3:**
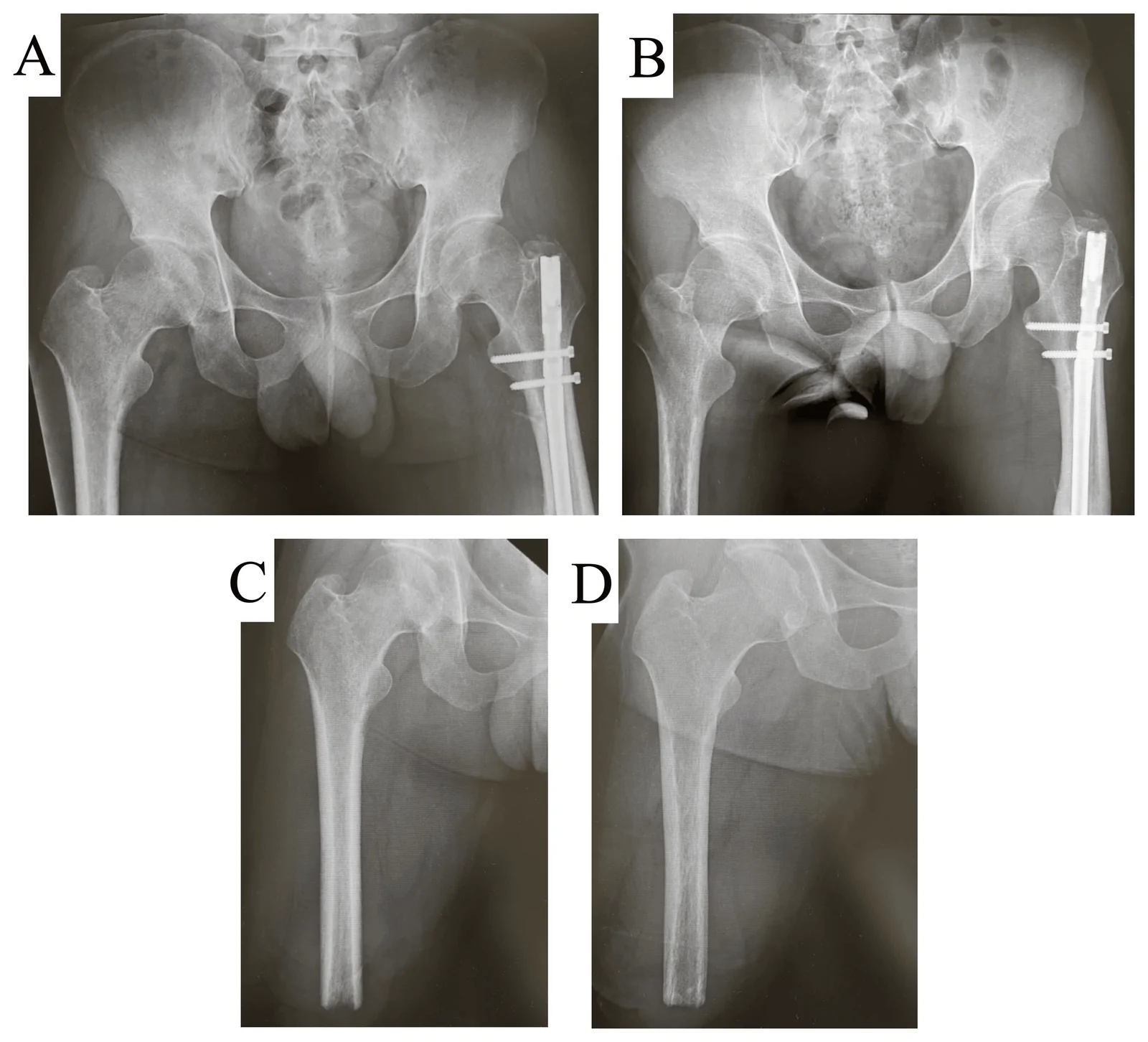
Plain radiographs of the amputated side obtained at the time of the first and second DXA examinations, three months apart, both before pharmacological treatment. (A, B) Anteroposterior views of both hips at the first and second examinations, respectively. (C, D) Anteroposterior views of the amputated-side femur, including the distal end of the residual limb, at the same two examinations. The right side is the amputated side; the intramedullary nail in the left femur is visible in panels A and B. BMD at the amputated total hip decreased by 20.1% between these two examinations. The later films suggest slightly increased radiolucency of the amputated-side proximal femur and reduced cortical thickness of the femoral shaft, but exposure conditions were not identical between the two examinations, so these observations are qualitative. BMD, bone mineral density; DXA, dual-energy X-ray absorptiometry.

Compared with the earlier films, the later films suggest slightly increased radiolucency of the amputated-side proximal femur and reduced cortical thickness of the femoral shaft. Because exposure conditions were not identical between the two examinations, these observations are qualitative.

In view of the rate and magnitude of the loss, treatment was discussed again, and the patient consented. Romosozumab 210 mg was administered subcutaneously once monthly for 12 months, from 9 to 20 months after amputation, and all 12 doses were given without interruption. Oral calcium and vitamin D supplementation was given concurrently and was continued throughout the subsequent treatment period. In Japan, romosozumab has been approved since January 2019 for osteoporosis at high risk of fracture, without restriction by age or sex, and denosumab and zoledronic acid are likewise approved for osteoporosis. All three agents were prescribed within their approved indications and reimbursed under national health insurance; no off-label treatment was given, although the licensed indication for romosozumab differs between countries. The decision was made by the attending physicians after discussion with the patient, who had declined treatment three months earlier and consented once the rate of loss had been documented; because the treatment lay within approved indications, it was not referred to an institutional conference. Teriparatide was considered as the alternative anabolic agent but was not selected, because the bone loss was concentrated at the proximal femur and the evidence for gains in proximal femoral BMD with teriparatide was judged weaker than that for romosozumab; an antiresorptive agent alone was considered unlikely to restore bone that was being lost at 20.1% per three months at the total hip. In accordance with the cardiovascular warning in the Japanese package insert, cardiovascular risk was assessed before treatment: there was no history of myocardial infarction, stroke, angina, or peripheral arterial disease, no hypertension and no dyslipidemia, no diabetes mellitus (HbA1c 5.6%), and normal renal function (eGFR 104 mL/min/1.73 m^2^); he neither smoked nor drank alcohol, and screening electrocardiography was normal. He was not considered to be at increased cardiovascular risk, and cardiovascular symptoms were enquired about at each monthly injection.

Sequential therapy and response

At the time of the twelfth and final romosozumab dose, 20 months after amputation, BMD had increased at all measured sites relative to the value at treatment initiation: +22.8% at the lumbar spine (0.976 g/cm^2^), +15.4% at the left femoral neck (0.682 g/cm^2^), +16.4% at the right femoral neck (0.554 g/cm^2^), +13.1% at the right trochanter (0.491 g/cm^2^), and +9.5% at the right total hip (0.586 g/cm^2^).

Because the gains achieved with romosozumab are not maintained without follow-on antiresorptive therapy, denosumab 60 mg subcutaneously was given at 21, 27, and 33 months after amputation, the first dose one month after the final romosozumab dose and the subsequent doses at six-month intervals. Treatment was then changed to zoledronic acid 5 mg intravenously, infused at 39 months, six months after the final denosumab dose and at the time the next denosumab dose would otherwise have been due; this interval was chosen to avoid a gap in antiresorptive coverage and the rebound increase in bone turnover that follows denosumab discontinuation. The timing was determined by this pharmacological consideration rather than by bone turnover markers. The change of agent was made to reduce the frequency of hospital attendance for a patient who had returned to full-time employment, and further annual infusions are planned, with reassessment of BMD and bone turnover at each.

At the final DXA examination, 33 months after the initiation of romosozumab and 42 months after the amputation, BMD had increased further at every site relative to the value at treatment initiation: +32.6% at the lumbar spine (1.054 g/cm^2^), +24.0% at the left femoral neck (0.733 g/cm^2^), +33.2% at the right femoral neck (0.634 g/cm^2^), +25.8% at the right trochanter (0.546 g/cm^2^), and +22.1% at the right total hip (0.653 g/cm^2^). Relative to the first DXA examination, however, recovery at the weight-bearing sites of the amputated side was incomplete: BMD of the right total hip remained 2.5% below the value recorded five months after the amputation, and that of the right trochanter exceeded it by only 7.5% (Table 3, Figure 2).

Bone turnover markers decreased progressively during treatment (Table 2 and Figure 4).

**Figure 4:**
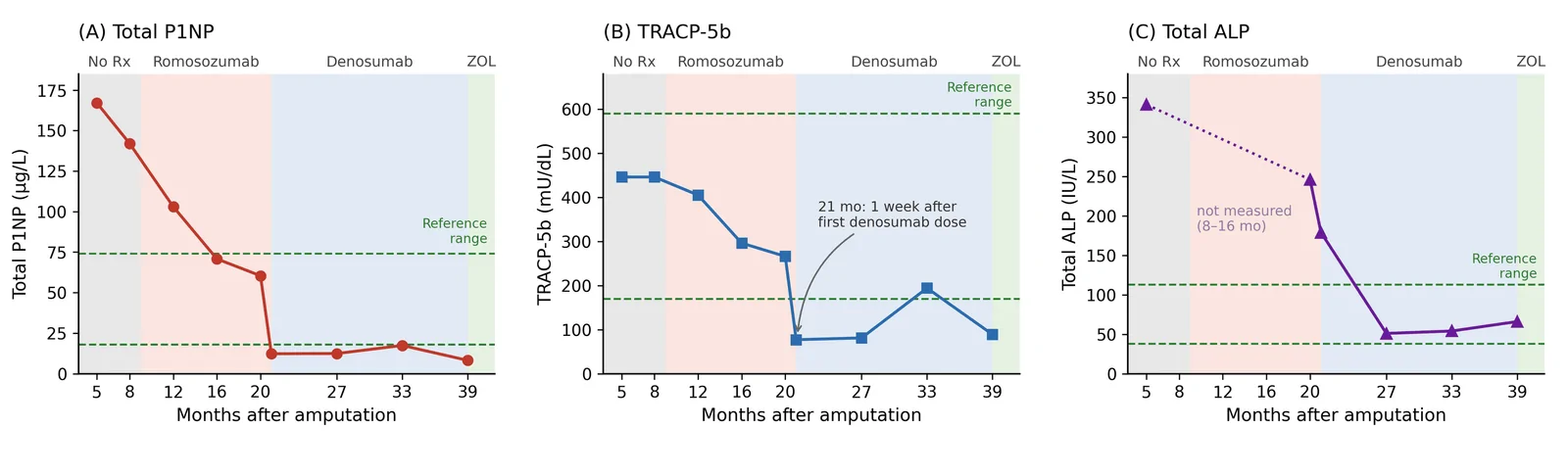
Serial bone turnover markers. (A) Total P1NP. (B) TRACP-5b. (C) Total ALP. The horizontal axis gives the time in months after amputation and is drawn to scale; the plotted points are the days on which blood was sampled, at 5, 8, 12, 16, 20, 21, 27, 33, and 39 months, which differ from the days on which densitometry was performed (Table 1, Figure 2). Samples were obtained on the day of each scheduled drug administration and, with one exception, immediately before the injection or infusion, so those values are trough values at the end of a dosing interval. The exception is the sample at 21 months, which was obtained one week after the first denosumab dose during routine monitoring for hypocalcaemia; the abrupt fall in TRACP-5b between 20 and 21 months reflects the rapid suppression of bone resorption that follows a first dose of denosumab. Dashed horizontal lines indicate the upper and lower limits of the reference range for each assay, and shaded bands indicate the treatment phases. Total ALP was measured with the same assay method throughout the observation period and was not measured at 8, 12, or 16 months; the dotted line in (C) indicates this gap. Total P1NP and total ALP were markedly elevated before treatment and decreased progressively during sequential therapy. The pre-treatment elevation cannot be attributed to a post-amputation skeletal response, because the left femoral shaft and pubic rami fractures were consolidating over the same period and bone-specific alkaline phosphatase was not measured. ALP, alkaline phosphatase; P1NP, N-terminal propeptide of type I procollagen; TRACP-5b, tartrate-resistant acid phosphatase 5b; ZOL, zoledronic acid.

Total P1NP decreased from 142 µg/L at treatment initiation to 8.2 µg/L at 39 months, on the day of the zoledronic acid infusion, TRACP-5b from 446 to 89 mU/dL, and total ALP from 341 IU/L before treatment to 66 IU/L. Serum calcium and inorganic phosphate remained within their reference ranges throughout, and renal function was preserved.

Outcome

Sequential therapy was tolerated. Romosozumab and denosumab caused only mild pain at the injection site. The first zoledronic acid infusion was followed by an acute-phase reaction, with fever to 38 °C and influenza-like symptoms. This possibility had been explained in advance. The reaction was treated with acetaminophen 2000 mg daily and resolved within one day, and the patient agreed to continue with the planned annual infusions. Serum calcium was measured one week after the first denosumab dose specifically to detect hypocalcemia and was 9.8 mg/dL. Over the whole observation period, serum calcium remained within the reference range at every measurement. The lowest value was 9.1 mg/dL with normal serum albumin, so the albumin-corrected value was unchanged, and no symptomatic hypocalcemia occurred. Calcium 600 mg and eldecalcitol 0.75 µg daily were given throughout the treatment period. Dental screening was performed before antiresorptive therapy was started and was normal, and no osteonecrosis of the jaw developed. No cardiovascular event, atypical femoral fracture, or fragility fracture occurred during the 42 months of follow-up, and renal function was preserved throughout (eGFR 80-104 mL/min/1.73 m²).

The patient continues to walk independently with his prosthesis indoors and outdoors without walking aids, wears the prosthesis for approximately 13 hours daily, and remains in employment.

## Discussion

Bone loss graded by loading condition

The pattern of bone loss in this patient was graded. Over the three months between the two untreated measurements, BMD decreased by 1.9% at the lumbar spine, 8.7% at the non-amputated femoral neck, 15.6% at the amputated femoral neck, and 20.1% at the amputated total hip. This ordering corresponds to the ordering of axial skeletal loading. The lumbar spine, which continued to be loaded during sitting and standing, was essentially preserved. The left femur, although fractured, internally fixed, and subject to marked disuse, was loaded through the foot once full weight bearing was permitted and showed an intermediate loss. Its fracture had not united at either measurement, and delayed union was diagnosed at the time of the second examination, so the limb was not being loaded as a normally healed femur would be. The amputated femur, which receives no direct axial load, showed the greatest loss. This distribution is consistent with the conclusion of the ADVANCE study that skeletal changes after amputation are predominantly mechanical in origin and represent localized unloading osteopenia [3] and with the observation that BMD deficits are greater after transfemoral than after transtibial amputation [1]. The near-preservation of the lumbar spine argues against a purely systemic process, but amputation also involves surgery, tissue trauma, inflammation, immobilization, altered gait, muscle atrophy, and neurovascular change, and a systemic contribution cannot be excluded on the basis of a single case.

A plausible mechanism is that the ischial ramal containment socket transfers body weight to the ischium and pubic ramus rather than through the femoral shaft, so that the residual femur receives the bending and torsional loads generated by the hip musculature but little axial load. Observations after transcutaneous osseointegration, in which the prosthesis is attached directly to the residual bone and skeletal loading is restored, support this interpretation. In a cohort followed for at least five years after single-stage press-fit osseointegration, all transfemoral amputees had local disuse osteoporosis before surgery, with a mean femoral neck BMD of 0.545 g/cm², and BMD of the amputated limb increased after osseointegration while that of the intact limb decreased [14]. The femoral neck values in the present patient, 0.564 g/cm² at the first measurement and 0.476 g/cm² three months later, lie in the same range. The comparison between the two limbs is therefore between two degrees of unloading rather than between an unloaded and a normally loaded femur.

Loss during intensive prosthetic gait training

The decrease occurred while the patient was progressing from wheelchair mobility to independent prosthetic gait. Mechanical loading was therefore increasing, not decreasing, during the interval in which proximal femoral BMD decreased by up to 20.1%. That bone loss is not reversed simply by becoming ambulatory has been shown before: in the prospective series of Bemben et al., hip areal BMD of the amputated limb decreased by 11-15% and had not recovered after 12 months of walking with a prosthesis [2]. Favorable local determinants did not protect this patient. Residual limb length and hip muscle strength have been identified as determinants of femoral neck BMD on the amputated side, with each additional centimeter of residual limb length corresponding to an increase of approximately 0.09 in T-score [15]; his residual limb measured 35 cm, and hip muscle strength was grade 4. The present case adds three elements to the existing evidence: the loss was documented at a single site with serial measurements at three-month resolution, its magnitude at the total hip exceeded the range previously described, and it occurred during a period of supervised, progressive gait training rather than during a period of inactivity.

The comparison with the transtibial case reported previously from the same institution, under the same rehabilitation approach, is relevant. That patient, a young man who undertook squats, rope skipping, and unilateral hopping on the prosthetic side, showed a 17.4%-24.3% increase in amputated-side hip BMD over three years [8]. The present patient wore his prosthesis for approximately 13 hours daily at a functional level corresponding to K3 and lost bone at the same sites. The difference in amputation level offers a direct explanation. After transtibial amputation, the femur remains within the load path and can be loaded axially through the residual tibia and the prosthesis, whereas after transfemoral amputation, an ischial containment socket transfers load to the pelvis and largely bypasses the femur. High-impact and multiplanar loading, which the Delphi consensus identifies as necessary components of an osteogenic exercise program [7], can therefore be delivered to the femur after transtibial but not readily after transfemoral amputation. These two cases together suggest that the achievable effect of loading-based intervention depends on amputation level and that after transfemoral amputation, pharmacological treatment deserves consideration when rapid loss is documented.

Comparison with two patients in whom bone loss was not measured

The clinical importance of detecting this decline is suggested, though not demonstrated, by two working-age men with transfemoral amputation reported separately from our institution [6]. Both had achieved independent prosthetic ambulation and returned to work, and both sustained an ipsilateral proximal femoral fracture after a minor indoor fall, at 16 months and six years after amputation. Neither underwent densitometry at any point, so their bone loss was never quantified, and neither received pharmacological treatment; radiographic assessment in those patients could show only qualitative side-to-side differences in cortical thickness and trabecular density. They were not controls, and their fracture outcomes cannot show that densitometry or treatment would have prevented fracture. The narrower point is that clinically important fractures do occur in this population, within the same interval after amputation in which the decline documented here took place, and that plain radiography is not an adequate substitute for densitometry: in the present patient, the films obtained at the two untreated examinations show no more than a suggestion of increased radiolucency and cortical thinning (Figure 3), whereas densitometry quantified the loss at the total hip as 20.1%, several times the least significant change corresponding to the minimum acceptable precision recommended by the International Society for Clinical Densitometry (ISCD) [16].

Rationale for and response to sequential pharmacological therapy

Romosozumab was selected because a sclerostin-directed agent has a specific rationale in this setting. Sclerostin, secreted by osteocytes in response to mechanical unloading, was elevated in the pre-ambulatory period after traumatic amputation and decreased thereafter, alongside elevation of bone ALP, in the prospective series cited above [2]. Total P1NP and total alkaline phosphatase were markedly elevated before treatment in the present patient, but these are not bone-specific measurements, and the left femoral shaft fracture was not united at either pre-treatment examination: delayed union was diagnosed and treated with LIPUS, and complete radiographic union was not confirmed until 11 months. An actively consolidating femoral shaft fracture is therefore the more likely explanation for the elevation of these markers, and because no stored serum remains, bone-specific alkaline phosphatase cannot be measured retrospectively to separate it from any post-amputation skeletal response. These markers are accordingly reported as descriptive data only and are not offered in support of the proposed mechanism. Sclerostin was likewise not measured, so the mechanistic rationale for sclerostin inhibition could not be verified in this patient, and the optimal timing of such treatment after amputation remains undetermined. Establishing whether a distinct post-amputation skeletal response exists would require prospective measurement of bone-specific markers from the time of amputation, ideally in patients without concomitant fractures.

Clinical precedent exists in spinal cord injury, the condition in which regional bone loss from mechanical unloading has been studied most closely. In 12 non-ambulatory women with chronic spinal cord injury, 12 months of romosozumab increased areal BMD by a median of 10.2% at the lumbar spine and 4.2% at the total hip, with no significant change at the distal femur or proximal tibia [10]; the response was graded by site in the same direction as here, being largest where mechanical stimulus was best preserved. In postmenopausal osteoporosis, corresponding 12-month gains were approximately 13% and 6% [17]. The larger gains in the present patient, +22.8% at the lumbar spine and +9.5% at the total hip, should not be read as evidence of greater efficacy. Age, sex, baseline bone turnover, concurrent fracture healing, loading pattern, and the technical context of densitometry all differ substantially between those populations and this case, and the comparison is offered only as hypothesis-generating context.

Two observations nevertheless argue against attributing the entire recovery to spontaneous resolution of the post-traumatic state. First, BMD was still decreasing steeply when treatment was started. Second, the increase was greatest at the lumbar spine, which had barely declined, and smallest at the amputated total hip, which had declined the most, whereas a recovery driven mainly by restored mechanical loading would be expected to show the opposite distribution. Neither observation excludes spontaneous stabilization, regression to the mean, or the effect of progressively increasing activity after return to work, and in an uncontrolled single case, the pharmacological contribution cannot be isolated.

Diagnostic and technical considerations

Anthropometric assessment requires care in this population. The uncorrected body mass index of 20.3 kg/m^2^ lies in the range at which low body mass index contributes to estimated fracture probability, whereas the amputation-corrected value of 22.0 kg/m^2^ does not; use of the uncorrected figure would have attributed to the patient a risk factor he did not have. T-score-based diagnosis is likewise not appropriate as the sole basis for assessment in a man of this age, and Z-scores were therefore used in parallel; the Z-score of the amputated total hip reached −3.0, below the expected range for age. 

DXA of the proximal femur on the amputated side is not standardized. Conventional positioning depends on internal rotation of the limb achieved through the foot, which is impossible after transfemoral amputation, and progressive muscle atrophy alters the soft-tissue composition within the region of interest. Institution-specific precision assessment had not been performed at our center, so the least significant change (LSC) for our densitometer could not be calculated. However, the LSC values corresponding to the minimum acceptable precision recommended by the ISCD are 5.3% for the lumbar spine, 5.0% for the total hip, and 6.9% for the femoral neck [16], and the changes observed here exceeded those thresholds by a wide margin. The intramedullary nail in the left femur precluded measurement of the left total hip and trochanter, so the comparison between limbs was restricted to the femoral neck.

Questions for prospective evaluation

The period of greatest fall risk after amputation coincides with the period of most rapid bone loss [2,4]. Femoral fracture in an amputee is most often sustained on the amputated side and carries higher mortality than hip fracture in a comparable non-amputee cohort [5]. The reported series consist largely of elderly patients [5], which may obscure the fact that proximal femoral fracture after a minor fall also occurs in working-age transfemoral amputees who walk independently with a prosthesis and are in employment [6]. In such patients, the consequences extend beyond mobility to the loss of employment that prosthetic rehabilitation had been directed at restoring. Despite this, densitometry is rarely performed after amputation; serial DXA is so uncommon that the timeline of bone loss has had to be reconstructed indirectly from computed tomography obtained for other purposes [18], and reduced BMD at the amputated-side hip persists for years, remaining evident in older amputees [9,19].

A single case cannot support a screening schedule or a treatment threshold, and neither the intervals nor the thresholds applied to this patient have been validated in this population. What the case does is define questions that prospective studies could answer. First, when should densitometry first be performed after major lower limb amputation, and how often should it be repeated during the first two to three years, given that the period of greatest fall risk coincides with the period of most rapid loss [2,4]? Second, does an interval decline exceeding a center-specific least significant change, or a proximal femoral Z-score below the expected range for age, identify patients in whom treatment alters the trajectory? Third, does such treatment reduce fall-related fractures, and does it preserve employment and community participation, rather than BMD alone? Answering these questions would require prospective cohorts with standardized serial densitometry of the lumbar spine and both hips, reporting of center-specific precision and positioning protocols, stratification by amputation level, residual limb length, socket design, and prosthetic use, and controlled evaluation of pharmacological treatment integrated with fall prevention, prosthetic optimization, and loading-based rehabilitation. For spinal cord injury, in which regional bone loss is likewise driven by mechanical unloading, the ISCD convened a task force and published official positions covering DXA-based diagnosis, fracture risk prediction, and monitoring of response to therapy [20]; no equivalent guidance exists for lower limb amputation, and the observations reported here indicate why developing it would require data of that kind.

One finding in this patient bears directly on the urgency of those questions. Even after 33 months of sequential therapy, which produced a 22.1% increase from the treatment nadir, BMD at the amputated total hip remained 2.5% below the value measured five months after amputation. Bone lost during this early window may not be fully recoverable.

Limitations

This is a single case without a control, and no causal inference can be drawn from it. Several specific limitations should be noted. First, no densitometry was performed at the time of injury, so the onset of bone loss and the true nadir are unknown, and the magnitude of loss during the first five months could not be quantified; the observed decrease of 20.1% represents only the portion of the loss that was captured. Second, the non-amputated limb was not an unaffected control, having sustained a femoral shaft fracture treated with an intramedullary nail that had not united at either pretreatment examination, and the nail additionally precluded measurement of the left total hip and trochanter. Third, serum 25-hydroxyvitamin D, intact parathyroid hormone, testosterone, and sclerostin were not measured, so vitamin D insufficiency and secondary hyperparathyroidism cannot be excluded as contributors, and the proposed mechanistic rationale for sclerostin inhibition could not be verified in this patient; the evaluation for secondary causes was therefore incomplete before the start of potent therapy, and these measurements cannot be added retrospectively because no serum was stored. Bone-specific alkaline phosphatase was likewise not measured, so the elevated bone formation markers cannot be attributed to a post-amputation skeletal response rather than to healing of the concomitant fractures, a limitation that applies to any patient in whom amputation and fracture arise from the same injury. Fourth, no protocol specific to the amputated limb was used for densitometry, and the position of the residual limb was not standardized between examinations; institution-specific precision assessment had not been performed, so the LSC could not be calculated for our densitometer, and the exact percentage changes should be read as approximations. Fifth, the functional level was estimated clinically as K3 rather than determined with a validated instrument, and prosthetic wearing time was recorded only during follow-up, not during the interval of rapid loss; physical activity was not quantified by accelerometry, so the contribution of progressively increasing mechanical loading to the recovery cannot be separated from the pharmacological effect. Regression to the mean, spontaneous stabilization of the post-traumatic state, and maturation of the residual limb cannot be excluded as contributors to the observed recovery. LIPUS was also applied to the left femoral shaft for delayed union during the first two months of romosozumab treatment; it was directed at the mid-shaft, remote from all five regions of interest, but it is a concurrent skeletal intervention and is reported for completeness. Sixth, bone quality was not assessed by trabecular bone score or high-resolution peripheral quantitative computed tomography, and BMD was used as a surrogate for fracture risk because no fracture occurred during follow-up. Seventh, the comparison drawn with two previously reported patients from the same institution [6] is a comparison between separate individuals reported at different times, not a controlled contrast, and it cannot demonstrate that densitometry or pharmacological treatment would have prevented their fractures. Finally, this patient was a young man with a traumatic amputation who achieved a high level of prosthetic activity, and the findings may not be generalizable to older patients with dysvascular amputation.

## Conclusions

Serial DXA in this patient documented a 20.1% decrease in BMD at the total hip of the amputated side over three months within the first year after traumatic transfemoral amputation, during intensive prosthetic gait training with progressive weight bearing. The magnitude of loss was graded according to loading condition, being least at the lumbar spine and greatest at the amputated proximal femur, a pattern compatible with a predominantly mechanical mechanism, although systemic contributions from trauma, inflammation, and immobilization cannot be excluded. BMD subsequently increased at every measured site during sequential treatment with romosozumab, denosumab, and zoledronic acid, but the amputated total hip did not regain the value recorded five months after amputation. Because this is a single uncontrolled observation in which fracture healing, continuing rehabilitation, and increasing physical activity coincided with pharmacotherapy, the recovery cannot be attributed to the medication. Together with a previously reported transtibial case in which high-impact training increased amputated-side hip BMD, these observations suggest that what loading-based rehabilitation can achieve for the femur depends on the level of amputation and that pharmacological treatment merits evaluation after transfemoral amputation. Bone loss of clinically relevant magnitude after transfemoral amputation is measurable and measurable early. Prospective studies are needed to determine whether early serial densitometry and treatment when rapid decline is identified reduce fragility fractures or improve clinically meaningful outcomes in this population.
